# Squamous cell carcinoma in a giant bladder diverticulum

**DOI:** 10.11604/pamj.2015.20.378.6765

**Published:** 2015-04-16

**Authors:** Yassine El Abiad, Fouad Bakloul

**Affiliations:** 1 My Ismail Military Hospital, Meknes, Morocco; 2 Avicenne Teaching Hospital, Rabat, Morroco

**Keywords:** Squamous cell carcinoma, bladder diverticulum, surgery

## Image in medicine

A 65-year-old man with a history of recurrent urinary tract infections presented after a 3-months of visible hematuria. Physical and laboratory examinations revealed anemia and impaired renal function. Ultrasound and magnetic resonnance imaging (MRI) showed an invasive bladder tumor developing in a large posterior diverticulum (A and B) with rectal deviation (A) and responsible of a left hydronephrosis. First, the patient underwent a blood transfusion and a left percutaneous nephrostomy. Two weeks later, we perfomed a cystoscopy and a transurethral resection of the tumor. Pathology revealed a locally advanced squamous cell carcinoma (SCC) (stage > p T2). The patient had no history of bilharziasis. Since the surgical extirpation was not possible, the patient was treated with chemoradiotherapy, but died 6 months later. Non-bilharzial SCC represents < 5% of vesical tumors, it is caused by chronic irritation of the urothelium and often diagnosed at an advanced stage. The intradiveticular location account for approximately 1% of all bladder tumors and make the prognosis poorer due to lack of muscle barrier. This case combine these two rares conditions.

**Figure 1:**
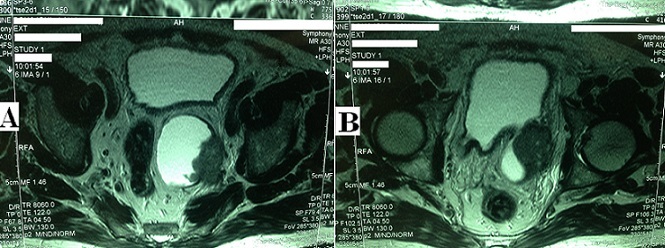
Squamous cell carcinoma in a giant bladder diveticulum

